# ‘Down to the person, the individual patient themselves’: A qualitative study of treatment decision‐making for shoulder pain

**DOI:** 10.1111/hex.13464

**Published:** 2022-03-15

**Authors:** Christina Maxwell, Karen McCreesh, Jon Salsberg, Katie Robinson

**Affiliations:** ^1^ School of Allied Health, Faculty of Education and Health Science University of Limerick Limerick Ireland; ^2^ Health Research Institute, Faculty of Education and Health Science University of Limerick Limerick Ireland; ^3^ Ageing Research Centre, Faculty of Education and Health Science University of Limerick Limerick Ireland; ^4^ School of Medicine, Faculty of Education and Health Science University of Limerick Limerick Ireland

**Keywords:** musculoskeletal, public and patient involvement, qualitative study, shoulder pain, treatment decision‐making

## Abstract

**Introduction:**

Many inconsistencies have been identified in the translation of evidence‐based treatment recommendations for musculoskeletal shoulder pain into healthcare services, with little known about factors influencing decision‐making. The objective of this study was to explore the views and experiences of healthcare providers (HCPs) and people living with shoulder pain on treatment decision‐making.

**Methods:**

Adopting a qualitative design, purposeful sampling was employed to recruit 13 individuals with nonspecific musculoskeletal shoulder pain and 30 HCPs. Data were collected through 1:1 semi‐structured interviews and analysed using an approach informed by Constructivist Grounded Theory. To facilitate analysis, two patient and public involvement (PPI) meetings were conducted.

**Results:**

Most participants (69%) had shoulder pain of ≥1‐year duration. Biomechanical beliefs about shoulder pain predominated and were heavily influential in decision‐making for both patients and HCPs. Despite a consensus that therapeutic alliance facilitated decision‐making, the extent of collaboration between HCPs and patients in treatment decision‐making was rather limited. In addition to condition‐specific factors, Individual patient characteristics and resources also influenced treatment decisions.

**Conclusion:**

Findings revealed the complexity of the decision‐making process for both patients and HCPs, exposing substantial gaps between the reported views and experiences of participants and the principles of client‐centred and evidence‐based practice. There is a pressing need to enhance the translation of evidence‐based knowledge into practice in this clinical area.

**Patient or Public Contribution:**

In line with a consultative approach to collaborative data analysis, a subgroup of participants attended two PPI meetings to provide commentary and feedback on preliminary findings.

## 1 INTRODUCTION

Shoulder pain is the third most common musculoskeletal condition presenting in primary care. It often evolves into a chronic condition, with over half developing persistent pain beyond 6 months.^1^ Musculoskeletal shoulder pain describes a spectrum of conditions, including subacromial pain syndrome, shoulder impingement, or rotator cuff disease.^2^ Current treatment recommendations commonly include analgesia (e.g., paracetamol and nonsteroidal anti‐inflammatory drugs), glucocorticoid injections and exercise therapy as first‐line options, with surgery considered a secondary intervention.^3^ Many inconsistencies have been identified in the implementation of up‐to‐date treatment recommendations for this population.^4^, ^5^, ^6^ Surgery has limited proven clinical benefit for full‐thickness rotator cuff tears,^7^ and is not recommended for subacromial pain syndrome or rotator cuff disease.^2^ This is unsurprising as structural shoulder pathology does not correlate with shoulder pain.^8^, ^9^ Nevertheless, shoulder surgery rates continue to increase.^6^

A growing number of qualitative studies have explored the experiences of people with shoulder pain and healthcare providers (HCPs) working with this cohort. A recent qualitative synthesis found that individuals with shoulder pain experience emotional, social and functional upheaval, express strong biomechanical beliefs about shoulder pain and are fearful of movement and exercise.^10^ A further qualitative synthesis demonstrated a lack of consensus amongst HCPs on how to manage shoulder pain, difficulties implementing research recommendations and challenges in getting patients to ‘buy in’ to exercise‐based treatment.^11^ This body of research provides further evidence of inconsistencies in the translation of evidence‐based recommendations into healthcare.

Shared decision‐making (SDM) is advocated to improve communication of healthcare options, facilitate improved quality of care and better implementation of evidence‐based recommendations.^12^ A limited body of research on shoulder pain has explored treatment decision‐making. Reported patient treatment priorities include a desire to regain movement, understand the problem and be cared for by someone who understands their condition.^13^ Patient decision‐making relating to surgery has also been explored, with failed nonsurgical treatment, pathoanatomical beliefs, limited information on treatment choices/risks and a strong preference for surgery, all increasing the likelihood of pursuing this option.^14^, ^15^ While many studies have explored HCP decision‐making, relatively few have focused on shoulder pain. One study of physiotherapists (PTs) revealed the influence of both expressed and unexpressed workplace norms, as well as clinical experience on decision‐making, sometimes over‐ruling research findings.^16^ Given the inconsistencies in the translation of evidence to practice and the limited attention to decision‐making to date, this study aims to explore the views and experiences of HCPs and people living with musculoskeletal shoulder pain on treatment decision‐making.

## 2 METHODS

### 2.1 Study design and ethics

This qualitative study was informed by Grounded Theory (GT) methodology, specifically the Constructivist Grounded Theory (CGT) approach, as described by Charmaz.^17^ GT methodology is a flexible methodology often used when there is little known about a phenomenon, where a theory is generated from data inductively with the aim of constructing an explanatory theory that reveals a process rooted in the area of inquiry.^17^ CGT differs from other GT approaches due to its focus on acknowledging multiple realities, engagement in critical analysis throughout the process and capacity for developing high‐level conceptual understanding.^18^ Hence, we aimed for a high‐level conceptual understanding of treatment decision‐making grounded in empirical data. This study adopts the consolidated criteria for reporting qualitative research (COREQ) (File S1).^19^ Ethical approval was granted by research ethics committees at three hospital sites and one sports surgery clinic in Ireland (Ref: C.A. 2251, Ref: 120/19, 30/10/19, Ref: SAREB201932).

### 2.2 Participants and recruitment

As Ireland has public (Health Service Executive) and private healthcare systems, using purposive sampling, recruitment took place across both public and private clinical sites. A gatekeeper at each site disseminated a recruitment pack to currently practising Orthopaedic (OC) and Rheumatology Consultants (RC), PTs, General Practitioners (GP) and Clinical Nurse Specialists (CNS) who treat people with shoulder pain. Using criteria reported in prior studies,^2^, ^20^ HCPs were invited to enrol as study participants and/or to distribute recruitment packs to patients meeting inclusion criteria based on those reported in prior studies, i.e. adults with ≥6‐week history of musculoskeletal shoulder pain, (see File S2). Given the associated greater morbidity levels and socioeconomic burden,^2^ individuals with persistent pain were recruited. Data were analysed in tandem with data collection. Recruitment was guided by theoretical sampling with efforts made to recruit individuals who could provide relevant data to develop the CGT based on initial analysis. Theoretical sampling was also pursued during the interviews with lines of inquiry being followed in later interviews based on concepts identified during concurrent data analysis of earlier interviews.^21^ The final sample size was determined at the point of theoretical saturation, the point in sampling where no new properties emerged and categories appeared to be ‘saturated’.^22^ Recruitment was affected by the outbreak of the COVID‐19 pandemic. In the final month of recruitment (March 2020) several HCP interviews were scheduled with little time between them as the interview team was concerned about the potential impact of the pandemic on future HCP participation. This somewhat limited concurrent data collection and analysis. Following preliminary analysis of these final interviews, it was determined that theoretical saturation was achieved. Subsequently, an additional 1–2 participants across stakeholder groups were recruited for confirmation purposes.^22^

### 2.3 Data collection

Data were collected by C. M. through 1:1 in‐depth semi‐structured interviews between 6 December 2019 and 26 March 2020, the preferred data collection method when using a CGT approach.^23^ The interview guides were developed based on the research objective and covered four key topics for HCPs: clinical experience, diagnostic confidence, treatment decision‐making and treatment outcomes, as well as five key topics for patients: personal experience, motivation to seek treatment, treatment expectations, treatment decision‐making and recovery expectations. To refine these guides, C. M. conducted a pilot interview with one HCP and one individual with shoulder pain, who were not subsequently included in the study. At each of the clinical sites, face‐to‐face interviews took place in a private room. Those unable to attend face‐to‐face interviews participated in telephone interviews. Interviews were audio‐recorded and fieldnotes were documented. Recordings were transcribed verbatim by a transcription service. Basic demographic information was collected from all participants (File S3). All participants had the opportunity to review transcripts before analysis.

### 2.4 Data analysis

Data were analysed using an approach informed by CGT. Transcripts were exported to NVivo (Version 12; QSR) and three key analytic techniques of coding, categorization and constant comparison were employed.^17^ Data from people with shoulder pain and HCPs were analysed separately and later mapped onto each other. Initially, C. M. coded all transcripts descriptively, later progressing to focused coding, which became more conceptual as the analysis progressed. Constant comparison of all data and codes across patient and HCP accounts was completed. This facilitated conceptualization of codes into higher‐order categories. Four categories were identified and further refined through constant comparison. Drafting analytic memos allowed a record of changes in interpretations of codes and furthered analysis at a conceptual level. Data were analysed by C. M. over a 12‐month period from the start of data collection, with regular meetings with K. R. and K. M. to challenge emerging interpretations.^24^

### 2.5 Public and patient involvement in data analysis

Following a consultative approach to collaborative data analysis, preliminary findings were presented to a subgroup of participants at two public and patient involvmenet (PPI) meetings in Novemeber 2020 (facilitated by C. M.) for commentray and feedback, in line with a consultative approach to collaborative data analysis.^25^ The use of PPI has been advocated to help correct misinterpretations and to challenge the way in which findings are reported.^26^, ^27^ For each meeting, 1–2 participants from each group were invited to participate. Meetings were one hour in duration, conducted via Microsoft Teams (V. 1.4.00.11161) and audio‐recorded but not transcribed. Attendees at the PPI meetings were largely in agreement with preliminary findings. One preliminary concept, ‘fear of making a mistake’, describing decisions to refer onwards, was challenged, with attendees clarifying that this was driven not by fear but as a ‘safeguarding’ mechanism (PT2), to ensure that nothing was missed. This refined interpretation was used in the later analysis when documenting beliefs relating to imaging and its role in decision‐making (Category 4). One person with shoulder pain described their prior expectations of a more hands‐on physiotherapy approach and how the detailed information given on treatment options enabled a ‘collaborative’ (P6) process. Also discussed was the role of trust in being open to exploring other treatment options. The centrality of trust to establishing a therapeutic alliance (TA) and its influence on treatment decision‐making was further confirmed by this discussion (Category 1).

### 2.6 Researcher positionality

CGA acknowledges that researchers cannot stand apart from the research process. Charmaz^28^ calls for methodological self‐consciousness where CGA researchers turn a deeply reflexive gaze back on themselves, the research process and the empirical world. Thus, we scrutinized our positions, privileges and priorities and assessed how they affected the research process and the relationship of the interviewer (C. M.) with participants. Data collection and analysis was completed by C. M. (Specialist Musculoskeletal Physiotherapist and PhD Candidate), with supervision, critical feedback and discussion with coauthors who have expertize in chronic pain and musculoskeletal conditions (K. R. and K. M.), physiotherapy (K. M.), occupational therapy (K. R.), social science (K. R. and J. S.), patient involvement in research and anthropology (J. S.), as well as from those in attendance at the PPI meetings. C. M. has over 10 years of experience in treating people with shoulder pain, as well as considerable experience conducting interviews and focus groups. Central to methodological self‐consciousness was the consideration of the physiotherapy backgrounds of C. M. and K. M. Through reflexive notes and research team discussions, the team's prior experiences, as well as personal and professional treatment biases, such as potential bias in favour of physiotherapeutic interventions, were considered.

Before study commencement, C. M. and K. M. were professionally acquainted with some HCPs who subsequently enrolled in this study but had no relationship with any of the patients recruited. All participants were informed that C. M. was a PT and that this study formed part of her PhD project. To minimize the potential impact of this, the interviewer (C. M.) engaged in frequent discussions with KR throughout data collection and recorded fieldnotes to support reflexivity. The phrasing of questions about physiotherapy and responses to participants' comments about physiotherapy during interviews were carefully considered by the interviewer.

### 2.7 Rigour

The study was conducted in line with guidelines on quality in CGT research.^18^ Critical feedback and guidance were provided by K. R., an experienced qualitative researcher with prior experience of GT and critical analyses. A transparent description of how this study was conducted is presented, with recruitment guided by theoretical sampling and saturation. Member‐checking enabled participants to confirm the accuracy of data collected. C. M. maintained detailed analytic memos to record changes in the interpretation of codes, while also seeking regular feedback from K. R. and K. M., helping to ensure interpretive rigour.^24^ The PPI meetings facilitated additional interpretive analysis and revision, helping to further enhance rigour.^29^

## 3 RESULTS

Forty‐three interviews were completed with thirteen people having shoulder pain and thirty HCPs (13 PTs, 6 GPs, 2 RCs, 6 OCs and 2 CNSs). Participant demographics and characteristics are presented in File S3. Of note, most participants (69%) had shoulder pain of ≥1‐year duration. Thirty‐four interviews were completed face‐to‐face and the remainder by phone. Interviews ranged in length from 12 to 53 min (average 32).

### 3.1 Findings

Four categories were identified but the development of a substantive theory integrating these categories was not achieved. The categories do however provide greater conceptual clarity in relation to the factors influencing treatment decision‐making for shoulder pain. Enhancing conceptual clarity without necessarily developing substantive theory is considered a satisfactory outcome for a GT study.^30^ HCPs and patients articulated a desire to build and maintain a TA characterized by the trust to support decision‐making (Category 1). Despite HCPs articulating a desire to establish a TA with patients, HCPs appraised patients beyond their shoulders, taking into consideration a wide range of characteristics, such as age and gender (Category 2), revealing potential assumptions and stereotyping of patients. In contrast to claims about working in collaboration, we found limited evidence of SDM. HCP‐led decision‐making was most common (Category 3). As HCPs were most commonly leading decision‐making, their beliefs concerning shoulder pain were very influential in treatment decision‐making (Category 4). Biomechanical beliefs about shoulder pain predominated across HCPs and patients. When HCPs held such beliefs, this in turn was reflected in the education and advice given to patients subsequently impacting their beliefs, expectations and engagement in treatment. Biomechanical beliefs about shoulder pain also impacted HCP decision‐making concerning imaging, treatments, and onward referrals.

### 3.2 Identified categories

#### 3.2.1 TA and the desire to build and maintain it influences treatment decision‐making

The HCP‐patient relationship was considered a strong influence on treatment decision‐making by both groups. Participants described rapport, trust and patient confidence in the HCP as essential components of a successful relationship, helping to facilitate decision‐making, promote positive patient expectations and improve treatment engagement. HCPs referred to this relationship as a TA.

Patients referred to various factors associated with HCPs they could trust, such as making a positive first impression, having greater clinical experience, providing clear guidance and feelings of being cared for;

*…from the very first day that I met with them, I just felt like I could trust them. I felt in their hands. I felt like I was being cared for*. (P12, m [male], 41–65 [age range])

Patients also described distrust of certain HCPs, including OCs and PTs, mostly based on previous negative experiences or preconceived assumptions about a particular profession. This affected the relationship, treatment decision‐making, as well as treatment engagement. One patient described their negative experience with physiotherapy, referring to the treatment as ‘very basic’ and not meeting their expectations to recover within 3 months, resulting in them subsequently disengaging and opting for surgery (P9, f, 65<).

HCPs felt TA was strengthened when they acknowledged patients' unique experience of pain, listened to concerns, provided clear explanations, offered ‘a plan B’ (PT 13, f, 41–65), were available as a ‘point of contact’ (CNS1, f, 25–40) and provided opportunities for follow‐up.

Various barriers to building a TA were identified, such as workplace requirements to employ too many questionnaires, not being able to ‘click with’ (RC1, f, 41–65) certain patients and difficulty developing a relationship with patients if the recommended treatment conflicted with guidance from another trusted clinician or with patient beliefs and expectations. In the latter situation, HCPs sometimes opted to refer onwards for imaging or surgical opinion, even if ‘not warranted’ (PT3, f, 41–65), so that the patients could feel ‘validated’ and had received ‘the full care that they should get’ (GP2, m, 41–65). Adopting this strategy was perceived to improve the alliance by providing reassurance, improving treatment buy‐in and helping foster positive expectations towards nonsurgical treatments.

While the majority of HCPs expressed a strong desire to meet patient expectations and maintain an alliance, there was also a clear desire from some HCPs for patients to agree with HCP preferences. A small group of HCPs, including GPs and PTs, adopted a ‘take it or leave it’ approach, advising patients to ‘go somewhere else’ (GP1, f, 25–40) should they wish to pursue an alternative treatment to their recommendation. Some PTs and GPs indicated that they aimed to foster patient trust to achieve patient compliance with HCP treatment preferences.

*…to get compliance and to get them to do it. They have to trust you or like you, don't they?* (PT8, f, 41–65)

When agreeing with patient‐led decisions they were not fully supportive of, PTs and GPs described a ‘compromise’ (GP6, m, 65<) for the purpose of maintaining an alliance in the longer term. When describing the decision to agree with a patient‐led decision, one GP reported:

*…I won't stick my feet on the ground because I know that they'll be other battles to be fought down the line in the greater scheme of things*. (GP 5, m, 25–40)

Likewise, GPs and PTs sometimes opted to provide treatment for short‐term pain relief that aligned with patients' expectations of ‘getting better now’ (GP3, m, 41–65), often in the form of a CSI or ‘hands‐on’ treatment, as opposed to the treatment they considered more evidence‐based. This is illustrated in the following quote from a PT describing their rationale for using manual therapy.

*…it works, like I can't tell you how exactly. And that's what they want when they come in*. (PT1, m, 25–40)

#### 3.2.2 Condition‐specific and circumstantial patient factors influence treatment decision‐making

Participants described a variety of ‘patient factors’ (OC4, 41–65) affecting treatment decision‐making. They considered not only the specific shoulder condition but also broader personal, social, lifestyle and contextual factors, with the final treatment decision boiling ‘down to the person, the individual patient themselves’ (PT10, f, 25–40) and their ‘circumstances’ (P3, f, 41–65).

Some factors related directly to an individual's shoulder pain and were perceived by HCPs to negatively impact recovery potential and suitability of certain treatments, including high levels of pain, chronicity, widespread pain, night pain, inability to participate in activities and severe pathology identified by imaging. Other, broader factors included advancing age, female gender, poor general health, mental health difficulties, unhealthy behaviours, inability to accept changes in function, passive coping styles, caregiving responsibilities, poor social support, inflexible work situation, inability to afford treatment, ongoing medico‐legal claim, living in a rural area, low education level, cognitive impairment, negative previous treatment experiences, low motivation to engage in treatment\recovery, low treatment compliance, delayed access to treatment and low recovery expectations.

*I've had some patients over the years and there's no doubt, the ones I've had to deal with who've come back with recurrence pain… they're all universally kind of middle or older women who seem to be still unhappy with their shoulder*. (OC 2, 41–65)

When considering surgical referral, GPs and PTs were more likely to refer those following trauma, reporting high levels of pain/night pain, physically active, younger, employed, with high expectations of recovery, pain negatively impacting ‘quality of life and their ability to work’ (GP5, m, 25–40), following a ‘good course of physio’ (PT9, f, 25–40), after trialling ‘multiple injections’ (PT9, f, 25–40) or with confirmation on imaging of a ‘full tear’ (GP4, m, 41–65). In contrast, for those presenting with a more ‘degenerative type’ age‐related pathology, OCs were perceived to be ‘less likely to go in and do something with them’ (PT5, m, 41–65). Patients who opted for surgery reported various factors influencing their decision, including good general health, younger age, difficulties coping with pain—‘I just couldn't stick the pain’ (P1, f, 41–65), being more physically active, being unable to engage in exercise/active pursuits, perceiving that conservative treatment ‘wasn't delivering’ (P11, f, 41–65) and expressing the feeling that there was ‘something not right’ (P2, m, 41–65) in their shoulder.

Patients' resources were often cited as a factor influencing decision‐making. Many HCPs working within both public and/or private sectors reported that private health insurance was influential as it facilitated quicker access to imaging and treatment. In particular, OCs, GPs and PTs noted frustration when trying to access imaging or treatment through the public system, with patients expected to face ‘long waiting lists’ (GP3, m, 41–65), resulting in many GPs advising patients to go privately if they want to ‘get this sorted’ (GP2, m, 41–65). Consequently, some of these HCPs described pre‐emptively making onward referrals, sometimes before it was clinically indicated, to act as a ‘safety net’ (PT4, f, 41–65) to avoid delays. However, quicker access to treatment was not always considered advantageous, with some PTs working within the private sector highlighting that this could lead to higher surgery rates—‘When you end up in front of an orthopaedic surgeon, you're more likely to have surgery’ (PT14, m, 41–65). For those PTs working in private practice, the fact that patients were ‘self‐funding’ (PT1, m, 25–40) treatment appeared to be a strong driving force behind decision‐making, wishing to avoid patient perception that they ‘wasted’ (PT1, m, 25–40) their money, resulting in earlier referral for imaging and surgical opinion, and delivering treatment that satisfied patient expectations, such as providing patients with ‘a bit of a rub’ (PT3, f, 40–65). From the patient's perspective, the ‘financial burden’ (P4, f, 41–65) and ‘huge costs’ (P2, m, 41–65) involved with various treatment options was undoubtedly a factor that also influenced their decision‐making. Some patients noted how ‘delighted’ (P2, m, 41–65) they were to have private health insurance as, without this, they ‘wouldn't be able to’ (P4, f, 41–65) afford physiotherapy treatment, or would have to wait ‘months, years possibly down the road’ (P2, m, 41–65) to access surgery.

#### 3.2.3 Limited patient involvement in treatment decision‐making

Participants described a wide spectrum of involvement in treatment decision‐making, including HCP‐led, patient‐led and SDM processes. HCPs largely perceived patients to be passive, preferring their doctor or HCP to take the lead in decision‐making.

*I'd say I would think there's only about 30 percent that have any interest in being involved in it*. (CNS 2, f, 41–65)

This view was echoed by many patients who were comfortable with HCPs taking control of treatment decisions, as illustrated in the following quote from a patient;

*If he said to me, ‘I'm going to sprinkle holy water on it’, I would have been happy with that as well. I didn't care*. (P12, m, 41–65)

A number of PTs and OCs who appeared to view themselves in a more ‘expert’ role noted advantages of HCP‐led decision‐making, enabling them to implement treatment they felt to be most appropriate. Some described their clinical expertize as a ‘lever’ (PT3, *f, 41–65*) that facilitated patient trust and therefore allowed HCP‐led decision‐making. In contrast, for CNSs, there appeared to be a greater tendency to defer decision‐making to the consultant. In situations where patients appeared reluctant to ‘buy‐in' to treatment, PTs, CNSs and GPs occasionally sought reinforcement from another HCP in an attempt to influence treatment choice. This is typified by one HCP who noted that patients might be more inclined to ‘hear it from the consultant’ (CNS2, f, 41–65).

In contrast, a limited number of HCPs' reported patients were increasingly seeking to take the lead and be ‘more involved in what's happening to them’ (RC1, f, 41–65). For some HCPs, this was preferable, wishing to ‘push the decision back to the patients’ (RC2, m, 41–65). To facilitate patient‐led decision‐making, some patients highlighted the importance of having a ‘clear picture’ (P6, m, 65<) of their options, thereby boosting their confidence to make their ‘own decision’ (P6, m, 65<). However, when patients expressed strong treatment preferences, considered to be inappropriate by some HCPs, several PTs and GPs perceived this as a threat to their autonomy, unwilling to be 'dictated to by a patient' (PT3, f, 41‐65). One CNS highlighted the potential negative implications this scenario could have on patient recovery;

*…They think they know better. You can tell them everything, but you know, in their head, all they see is surgery… They've made a decision and no matter what you say, they've made a decision*. (CNS2, f, 41–65)

Many HCPs also described a preference towards an SDM approach, that is, including the person with shoulder pain as an equal partner and working ‘together towards making a decision’ (OC1, 41–65) as achieved through outlining all the options available, providing sufficient information, debating ‘the risks and the benefits’ (PT14, m, 41–65) of each, while also being transparent about the professional treatment biases that exist;

*…of course I'll have a bias, these are my tools, the surgeons will have the same because that's their tools*. (PT 11, f, 25–40)

HCPs described various strategies to confirm patient understanding and facilitate an SDM approach, achieved by asking the patient to relay their understanding, summarizing and checking—‘are we on the same hymn sheet here?’ (GP5, m, 25–40), dictating letters with the patient present, allowing time for questions in an uninterrupted environment, checking patient contentment at various intervals, encouraging attendance of family members, as well as providing educational resources. However, many GPs, OCs and PTs admitted that they did not routinely utilize such strategies, mainly due to time constraints.

Despite many HCPs describing patient involvement in decision‐making, few patients described an SDM process, with some recounting treatment being ‘done’ (P1, f, 41–65) to them, and in one case reflecting that they ‘wouldn't rush into it [the decision to have surgery] again’ (P1, f, 41–65). Patients' interpretations of a ‘joint decision’ varied. While some noted it had been a shared process, others did not feel ‘capable’ (P7, m, 41–65) of making the decision independently. For a small number of patients, a shared or ‘collaborative’ process was described;

We're beginning to talk about maybe other options… We're doing that in a collaborative way… He leaves it as much to my own intuition and my own um, you know, me knowing my own body best of all, I suppose. Um, so it's kind of teamwork. (P6, m, 65<)

#### 3.2.4 Beliefs about the cause of shoulder pain influence treatment decision‐making

Participants' beliefs about the cause of shoulder pain reflected a largely biomechanical understanding, directly affecting treatment decision‐making by influencing the education provided to and sought by patients, the perceived importance of imaging findings, and opinions on the treatment required.

When educating patients on the cause of their symptoms, many of the OCs, and to a lesser extent, GPs, had a tendency to describe the pain as resulting from specific anatomical ‘structural damage’ (OC5, 41–65) or anomaly, such as ‘a bony spur’ (OC1, 41–65), or that their ‘letterbox [subacromial space] is closed’ (GP6, m, 65<) or ‘tight’ (OC3, 41–65). In contrast, the other HCP groups typically adopted a nonspecific approach, attributing symptoms to ‘wear and tear’ (CNS1, f, 25–40) or age‐related ‘degenerative changes’ (PT6, m, 25–40), with few HCPs highlighting to patients that such changes are also seen in ‘asymptomatic patients’ (PT6, m, 25–40). The views and understanding of people with shoulder pain echoed these descriptions, with references to their shoulders being ‘damaged’ (P6, m, 65<) or ‘wear and tear’ (P13, f, 65<) being common. Irrespective of how ‘specific’ HCPs were when educating patients on the causation of symptoms, most used ‘visual and tactile things’ (OC6, 25–40) to educate patients and guide decision‐making, such as skeletons, anatomical images of ‘what a tear looks like’ (CNS2, f, 41–65), as well as analogies, for example, ‘like a truck going under a low bridge and it's scraping off the bridge’ (OC3, 41–65), with virtually no reference to psychosocial factors influencing shoulder pain.

Many participants referred to imaging as the ‘first port of call’ (P6, m, 65<), and an essential element of their decision‐making, providing an opportunity to see the ‘damage that had been done’ (P2, m, 41–65) and to ensure they ‘didn't miss anything major’ (PT4, f, 41–65). Imaging was an integral part of the decision‐making process for most patients, especially for those opting for surgery; ‘I'd definitely have the MRI and on that then, I would decide’ (P11, f, 41–65), with only a few describing an indifference towards referral for imaging. HCPs from within each group noted the perception that patients placed greater ‘faith’ in imaging recommendations over clinical opinion, with mainly RCs, GPs and PTs reporting difficulty explaining to patients that imaging findings ‘mightn't necessarily be the cause of your symptoms’ (PT1, m, 25–40), with one RC even suggesting it to be ‘a great waste of money’ (RC2, m, 41–65).

Biomechanical beliefs were also evident in the rationale provided for various treatments. Most GPs associated pain with inflammatory processes and, as a result, were ‘mostly prescribing nonsteroidal anti‐inflammatories’ (GP2, m, 41–65). The rationale provided by PTs for various treatment approaches reflected similar biomechanical understandings, referring to the ‘synthetic anti‐inflammatory’ (PT1, m, 25–40) effect of ultrasound, the ability of manual therapy to ‘free’ (PT1, m, 25–40) up movement restrictions and ‘relax’ (PT1, m, 25–40) tight muscles, as well as dry needling ‘to free out the muscles’ (PT12, f, 25–40). Many patients' views on physiotherapy echoed similar biomechanical views and a degree of scepticism, questioning how an exercise could ‘cure’ (P7, m, 41–65) ‘wear and tear’ (P7, m, 41–65).

When considering the use of CSIs, the rationale provided by HCPs mostly centred on its analgesic effects, providing ‘a way of fixing it now’ (CNS1, f, 25–40), enabling muscles to ‘fire more effectively’ (OC2, 41–65) and providing ‘a pain‐free window’ (PT6, m, 25–40) for rehabilitation. Many patients believed that CSIs were not a cure or long‐term solution, referring to HCP advice that they ‘can't guarantee that it mightn't need surgery in the future’ (P12, m, 41–65).

A biomechanical understanding was also revealed in decision‐making about surgery. For most OCs, surgery recommendations were based on the perception that ‘abnormal’ anatomical findings, such as a ‘hypertrophied’ (OC3, 41–65) acromial process, were the source of symptoms. Many patient accounts on why they were recommended surgery reflected such views;

…he was after telling me that I had this bony outcropping on the top of the shoulder, that, um, explained why no amount of injections or physio was going to fix that. Short of getting in there and removing it, it wasn't going to sort that one out. (P7, m, 41–65)

While biomechanical understanding predominated, other views were also articulated. Some OCs expressed a preference to ‘avoid getting someone on a conveyer belt towards surgery just because they have a tear’ (OC1, 41–65). Many HCPs described challenges trying to ‘convince’ (PT10, f, 25–40) patients that surgery is not required. In an attempt to reduce the fear associated with exercise, some PTs reported that they try to change patients' biomechanical understanding through education and reassurance such as ‘exercise that hurts doesn't always mean harm’ (PT8, f, 41–65).

## 4 DISCUSSION

### 4.1 Summary of key findings

This qualitative study explored the views of HCPs and people living with shoulder pain on treatment decision‐making. Biomechanical beliefs predominated and were influential in decision‐making for both patients and HCPs. While there was good agreement that TA facilitated treatment decision‐making, the extent of collaboration between HCPs and patients was limited. Individual patient characteristics and resources as well as condition‐specific factors also influenced decisions.

### 4.2 Comparison with existing literature

Participants in this study generally reported limited patient involvement in treatment decision‐making. An SDM approach is the preferred model for treatment decision‐making, shown to improve patient outcomes and reduce healthcare costs.^31^, ^32^ SDM is described variably as a joint process where clinicians and patients make decisions together,^12^ or as a continuum, where the extent to which the patient‐ and clinician‐lead decision‐making varies depending on patient preference and the context.^33^ Although numerous studies indicate that most patients seek to share decision‐making,^34^, ^35^ few patients within the current study described a shared approach. In some cases, HCPs associated a more passive approach with older patients, echoing the findings of previous studies and potentially reflecting ageist views of older adults as being less able to participate in decision‐making.^36^ We found little evidence of patients being encouraged to adopt a more active role,^37^ or of HCPs effectively communicating research evidence in a ‘clear, understandable and non‐misleading manner’ to support SDM.^38^

As shoulder pain is a multidimensional condition associated with a multitude of nonbiological factors,^39^ a biopsychosocial approach to its management is recommended.^40^ The use of a biopsychosocial approach for back pain has spurred the use of interventions, such as cognitive behavioural therapy (CBT)^41^ and pain neuroscience education.^42^ Despite a biopsychosocial approach being recommended for shoulder pain, we found that biomechanical belief predominated as has been previously reported.^10^, ^11^ Few participants described a holistic approach to treatment, with no mention of interventions such as CBT. Our findings show how biomechanical beliefs initiate a cascade of treatment decision‐making in terms of imaging decisions, intervention decisions and referral decisions and how such beliefs have an impact on how HCPs educate and advise patients, thereby influencing their beliefs and treatment expectations. The treatment decision‐making arising from such beliefs is inconsistent with the best available research evidence.^8^, ^9^

Patients' preferences are just as important as research and clinical expertize in treatment decision‐making.^43^ However, we found in most cases that HCP beliefs and clinical expertize far outweighed research evidence or patient preference. Some HCPs described the use of their ‘expert’ title as a ‘lever’ to foster compliance, irrespective of whether the proposed treatment reflected up‐to‐date recommendations. The way in which information is provided to patients heavily influences treatment decision‐making in relation to surgery.^44^ We found little evidence that HCPs consistently provide patients with balanced up‐to‐date information on the associated risks and/or benefits of different treatment options. Furthermore, our findings highlight the disparity between the private and public health systems in relation to expedient access to treatment, inappropriate referral and access to treatment incongruous with evidence‐based treatment hierarchy. Such inappropriate specialist referrals and underutilization of appropriate treatment for rotator cuff disorders have been described elsewhere.^45^

Both patients and HCPs emphasized the importance of TA in treatment decision‐making. The ‘active’ components of a TA are ‘empathy, congruence and unconditional positive regard’.^46^ Additionally, a TA can facilitate patient‐centred care.^47^ Although HCPs acknowledged the importance of a TA, descriptions of various patient factors influencing decision‐making are incongruent with the tenets of a TA. Some HCPs expressed negative expectations in relation to the engagement and recovery of women, those with chronic or widespread pain, as well as those involved in medico‐legal claims. While some of these factors have been identified as negative prognostic indicators for shoulder pain,^48^, ^49^, ^50^, ^51^ it is important that HCPs do not perpetuate or reinforce stereotypes in clinical practice. Adopting a subjective approach can negatively impact the quality, consistency and accuracy of decision‐making.^52^ While TA was valued by HCPs, our findings revealed it is not being fully realized in practice, with negative consequences for patient involvement and the potential for clinical biases to adversely influence treatment decision‐making.

### 4.3 Strengths and limitations

To our knowledge, this is the first qualitative study to explore the views of both people with shoulder pain and HCPs in relation to treatment decision‐making. Strengths of the study include the recruitment strategy involving recruitment at four clinical sites, within both private and public healthcare settings, including a large number and broad range of highly experienced HCPs and patients who had experiences of a variety of treatments, with attention to both theoretical sampling and saturation. Data were subjected to prolonged analysis and PPI consultation during analysis, thereby contributing to the study rigour.^29^ Although each stakeholder contributed to discussions during the PPI sessions, formal training may have further enhanced their participation, confidence and contributions.^53^ A further limitation of this study is that data collection and analysis were completed by a PT, thereby potentially influencing the responses collected and the analysis as such. Reflexivity, facilitated through fieldnotes and research team discussions, aimed to minimize this potential impact. While the interviewers' background may have influenced participants' responses, the data collected included wide‐ranging experiences of physiotherapy, both positive and negative. Furthermore, the involvement of participants in the interpretation of data collected and the refinement of identified categories was a further strategy to minimize the impact of the research team on the results and conclusions drawn. People with ≥6 weeks' history of musculoskeletal shoulder pain were eligible to participate. However, most participants (69%) had shoulder pain of ≥1‐year duration, with only one participant reporting symptoms of ≤3 months. Although people with shoulder pain discussed decision‐making across the entire period from symptom onset, the experience of those whose symptoms resolve within 3 months is not well represented in this study.

### 4.4 Implications for clinical practice and future research

The findings of this study can be used to support an SDM approach and evidence‐based treatment decision‐making in shoulder pain. It is recommended that future research on this topic should adopt a PPI approach from conception to dissemination as this would likely facilitate improved stakeholder engagement and enhanced application of findings in clinical practice.^54^ Future exploration of factors influencing patient's treatment decision‐making in the early phase after shoulder pain onset is warranted.

## 5 CONCLUSION

This qualitative study exploring the views of HCPs and people with shoulder pain on treatment decision‐making found that HCPs and patients articulated a desire to build and maintain a TA characterized by trust to support decision‐making. However, HCPs appraisal of patients revealed potential assumptions and stereotyping of patients in contrast with the tenets of TA. Limited evidence was found of SDM. Biomechanical beliefs about shoulder pain predominated across HCPs and patients. Such beliefs initiated a cascade of treatment‐related decisions. Findings indicate a pressing need for improved use of research evidence and enactment of TA combined with SDM in services for people with shoulder pain.

## CONFLICTS OF INTEREST

The authors report no conflict of interest. The Irish Research Council, which provided funding for this study, had no involvement in the study design; in the collection, analysis and interpretation of data; in the writing of the manuscript; or in the decision to submit this article for publication.

## Supporting information

Supporting information.

Supporting information.

Supporting information.

## Data Availability

The data that support the findings of this study are available on request from the corresponding author. The data are not publicly available due to privacy or ethical restrictions.
