# Supplementary material for: ‘Down to the person, the individual patient themselves’: A qualitative study of treatment decision‐making for shoulder pain
Source: Health Expect. 2022 Mar 15;25(3):1108–17. doi: 10.1111/hex.13464 (PMC9122451; doi:10.1111/hex.13464)
Supplement: Supplementary file 1 — Supporting information. [file HEX-25--s001.docx]

**Supplementary File 2. Patient Eligibility Criteria Information**

|  | - Inclusion criteria | - Exclusion criteria |
| --- | --- | --- |
| Individuals with shoulder pain | - ≥ 18 years of age - ≥ 6 week history of shoulder pain - Diagnosed with non-specific musculoskeletal shoulder pain (i.e. including rotator cuff-related pain (rotator cuff disease, tendinopathy, tendinosis, and partial-thickness rotator cuff tear), subacromial impingement syndrome) by a health care provider. - Able to communicate their experience and opinions in English and be articulate and clear. - Willing and able to consent. | - Full-thickness rotator cuff tear   i.e. a complete tear of two or more tendons.   - Recent history of a shoulder fracture or dislocation (i.e. in the last 6 months). - Shoulder pain induced by cervical spine movement - Shoulder pain of non-musculoskeletal origin, i.e. gastrointestinal (e.g. gall stone disease), neurological (e.g. cervical radiculopathy, peripheral nerve injury), cardiological (e.g. pulmonary embolism, myocardial ischaemia) or rheumatological diseases (e.g. Rheumatoid Arthritis). - Cancer or stroke-related shoulder pain. - Atraumatic shoulder instability - Adhesive Capsulitis (Frozen shoulder) - Severe glenohumeral osteoarthritis |
