# Supplementary material for: ‘Down to the person, the individual patient themselves’: A qualitative study of treatment decision‐making for shoulder pain
Source: Health Expect. 2022 Mar 15;25(3):1108–17. doi: 10.1111/hex.13464 (PMC9122451; doi:10.1111/hex.13464)
Supplement: Supplementary file 2 — Supporting information. [file HEX-25--s002.docx]

**Supplementary File 3. Table 1 & 2 Illustrating Patient and HCP Demographics and Characteristics**

| Table 1. Patient demographics and characteristics (n=13) | |
| --- | --- |
| Variable | Value N (% of sample) |
| **Age range**  40-64  65+  **Gender**; female/male  **Educational Level**  None  Secondary  Third Level  **Current Employment**  Full-time/Part-time  Retired  Self-Employed  Leave of Absence  Homemaker  Unable to work  **Current or previous Occupational Activities**  Manual Labour  Office-Work  Transportation  Hospitality/Retail  Healthcare  Education  Not applicable  **Employment Location;** Rural**/**Urban  **Duration of shoulder pain**  6-12 weeks  3-6 months  1-5 years  >5 years  **NRS**  0-3  4-6  7-10  **Healthcare providers**  GP  Physiotherapist  Orthopaedic Consultant  Other  **Treatments received**  Advice/education on pain management  Pain relief/Analgesia  Exercise  Manipulation/Mobilisation  Injections (range/average)  Massage  Electrotherapy  Surgery  Other | 8 (62%)  5 (38%)  9 (69%)/4 (31%)  1 (8%)  3 (23%)  9 (69%)  3 (23%)/ 4 (31%)  4 (31%)  1 (8%)  1 (8%)  1 (8%)  2 (15%)  2 (15%)  3 (23%)  1 (8%)  2 (15%)  3 (23%)  5 (17%)  1 (8%)  8 (62%)/5 (38%)  1 (8%)  3 (23%)  7 (54%)  2 (15%)  6 (46%)  3 (23%)  4 (31%)  10 (77%)  13 (100%)  8 (62%)  2 (Physical Therapist) (15%)  6 (46%)  5 (38%)  12 (92%)  5 (38%)  5 (1-6/3) (38%)  6 (46%)  None  7 (54%)  4 (2 x Dry Needling, 2 x MRI scan) (31%) |

| Table 2. HCP demographics and characteristics (n=30) | |
| --- | --- |
| Variable | Value |
| **Age range**  25-40  40-65  65+  **Gender**; female/male  **Educational Level**  Undergraduate  Masters  Doctoral  Postdoctoral  Other  **Member of shoulder specialist group**  ISES  ISERS  SCoP  EUSSER  BESS  ASES  Other  **Experience treating MSK shoulder pain**  3-5 years  5-10 years  10+ years  **Employment status**  Full-time/Part-time  **Employment setting**  HSE Hospital/ HSE Community  Private Hospital/ Private practice/clinic  Education  Research  Other  **Employment Location**; Rural/Urban  **Treatments used/indicated**  Advice/Education  Analgesia  Exercise  Joint manipulation  Joint mobilisation  Joint injections (average administered)  Massage  Electrotherapy  Surgery  Other  **Educational Resources**  Research Journals  Books  Social Media   - Blogs - Podcasts - Twitter   Other  Professional websites   - ICGP - ISCP - IITOS - Other   Shoulder specific websites   - ISERS - ISES - SCoP - EUSSER - BESS - ASSET   Conferences  Clinical Practice Guidelines | 12 (40%)  17 (57%)  1 (3%)  18 (60%)/12 (40%)  12 (40%  16 (54%)  2 (6%)  2 (6%)  1 (MB) (3%)  10 (33%)  6 (20%)  3 (10%)  2 (6%)  1 (3%)  1 (3%)  1 (Irish Society of Rheumatology) (3%)  3 (10%)  1 (3%)  26 (87%)  29 (97%)/ 1 (3%)  10 (33%)/ 3 (10%)  10 (33%)/ 11 (37%)  4 (13%)  2 (6%)  1 (Sports setting) (3%)  4 (13%)/26 (87%)  30 (100%)  17 (57%)  30 (100%)  4 (13%)  11 (37%)  16 (2-3) (53%)  10 (33%)  2 (6%)  10 (33%)  4 (13%) (1 Acupuncture, 2 onwards referral, 1 Taping)  27 (90%)  13 (43%)  12 (40%)  5 (17%)  14 (47%)  6 (20%)  3 (10%) (Instagram, meetings, Orthobullets)  7 (23%)  9 (30%)  2 (6%)  1 (3%) (ISR – Irish Society of Rheumatology)  6 (20%)  9 (30%)  4 (13%)  2 (6%)  8 (27%)  1 (3%)  13 (43%) (ISES, BESS, IITOS, ISERS, EULAR, ACR, ISR, SECEC, ASES, ASSSM, EUSSAR, FSEM)  6 (20%) |
